# N-Phenacylthiazolium Bromide Reduces Bone Fragility Induced by Nonenzymatic Glycation

**DOI:** 10.1371/journal.pone.0103199

**Published:** 2014-07-25

**Authors:** Brian S. Bradke, Deepak Vashishth

**Affiliations:** Department of Biomedical Engineering, Rensselaer Polytechnic Institute, Troy, New York, United States of America; University of Notre Dame, United States of America

## Abstract

Nonenzymatic glycation (NEG) describes a series of post-translational modifications in the collagenous matrices of human tissues. These modifications, known as advanced glycation end-products (AGEs), result in an altered collagen crosslink profile which impacts the mechanical behavior of their constituent tissues. Bone, which has an organic phase consisting primarily of type I collagen, is significantly affected by NEG. Through constant remodeling by chemical resorption, deposition and mineralization, healthy bone naturally eliminates these impurities. Because bone remodeling slows with age, AGEs accumulate at a greater rate. An inverse correlation between AGE content and material-level properties, particularly in the post-yield region of deformation, has been observed and verified. Interested in reversing the negative effects of NEG, here we evaluate the ability of n-phenacylthiazolium bromide (PTB) to cleave AGE crosslinks in human cancellous bone. Cancellous bone cylinders were obtained from nine male donors, ages nineteen to eighty, and subjected to one of six PTB treatments. Following treatment, each specimen was mechanically tested under physiological conditions to failure and AGEs were quantified by fluorescence. Treatment with PTB showed a significant decrease in AGE content versus control NEG groups as well as a significant rebound in the post-yield material level properties (p<0.05). The data suggest that treatment with PTB could be an effective means to reduce AGE content and decrease bone fragility caused by NEG in human bone.

## Introduction

Non-traumatic skeletal fractures are directly related to increased incapacitation, morbidity, and mortality and pose a serious health problem to an aging population [1]–[3]. Several age-related changes in bone morphology and composition have been identified and were subsequently linked to an increased risk of non-traumatic fracture [4]–[7]. One such change is the accumulation of advanced glycation end-products (AGEs) within the collagen network of cortical and cancellous bone [8]–[10].

AGEs are a series of post-translational modifications in the cross-link profile of long-lived proteins throughout the body [11]. These modifications are caused by the reaction of reducing sugars found in the extracellular space with amino groups of collagen through a process called non-enzymatic glycation (NEG). The resulting covalent, glucose-derived protein crosslinks are naturally occurring and can be replicated in-vitro by subjecting collagen to reducing sugars in solution [12].

Because collagen is a structural protein, altering its crosslink profile impacts the form and function of the constituent tissue. Bank et al. demonstrated that in-vivo NEG produces a stiffer organic matrix in normal human cartilage [12]. The biomechanical properties of human bone, which has an organic phase consisting primarily of type I collagen, are similarly affected by NEG [13], [14].

The organic phase of bone is predominately responsible for the tissue's ductility and overall ability to absorb impact loading as it allows bone to deform and release energy before failure. Stiffening of the collagenous matrix due to NEG reduces the total strain the tissue is able to resist before ultimate failure. This reduction is measured as a decrease in post-yield and ultimate strain values via mechanical testing. Vashishth et al. [14] demonstrated that changes in bone quality resulting from NEG had a significant effect on post-yield properties and the tissue's overall ability to resist fracture. We have also demonstrated that AGEs in bone accumulate at an increased rate during bisphosphonate therapy for post-menopausal osteoporosis and this accumulation of AGEs is associated with changes in post-yield fracture properties [15].

Knowing that NEG contributes to increased bone fragility and increased fracture risk, we set out to identify a compound that cleaved the established AGE crosslinks in bone. In a 1996 publication, Vasan et al. reported that a novel, thiazolium-based nucleophile called “N-Phenacylthiazolium Bromide” (PTB) selectively cleaves AGE crosslinks in rat-tail tendon both in-vitro and in-vivo [16].

In particular, they administered PTB to Lewis rats with above-average AGE content due to laboratory-induced diabetes. Dissection and collagen extraction from the tail-tendon revealed a decrease in AGE crosslinks after 32 weeks of treatment, proving the feasibility of in-vivo treatment [16]. Our study expands upon their groundbreaking work in rat models, by treating human skeletal tissue with PTB in vitro and collecting mechanical data in addition to biochemical analysis. Bolstered by this previous research here we evaluated the effectiveness of PTB in reversing the effects of NEG on human cancellous bone in vitro.

## Materials and Methods

Cancellous bone cylinders were taken from the tibial plateaus of 9 male cadavers aged 19, 29, 39, 45, 48, 49, 50, 64 and 80. None of the donors were diagnosed with osteoarthritis, and they were also certified to be free of metabolic bone diseases, HIV, and hepatitis B (National Disease Research Interchange and International Institute for the Advancement of Medicine). No live human subjects were involved in this research study (IRB Waiver, Albany Medical College Hospital/Rensselaer Polytechnic Institute). Cadaveric human specimens used in the study were obtained the anatomical gift registry (National Diseases Research Interchange, Philadelphia PA).

The cylinders were obtained under wet-conditions using a three-eighths inch diameter, diamond-tipped, core drilling bit (Starlite Industries, Inc., www.starliteindustires.com) that was mounted in a standard bench-top drill press. The cylinders were then wet-machined to a specific length of ten millimeters using a variable speed diamond saw from Buehler Inc. (www.buehler.com). Specimens were excised from the donor's centralized tibial plateau, parallel to the longitudinal axis of the diaphysis, and were randomly assigned to the treatment groups described below.

A total of eighteen cylindrical specimens were obtained from each of the nine donors. Each specimen was thoroughly rinsed with and stored in normal calcium-buffered saline at −20°C until testing. Previous studies done in our laboratory have demonstrated that this procedure preserves the mineral and organic matrix within the bone [14], [17].

The bone specimens of each donor were randomly assigned to one of six treatment groups. The treatment groups consisted of a control (C), a glycated (ribosylated) control (R), and four treatment groups (X1-X4), each with a different concentration of PTB in a phosphate buffered saline solution. The specific treatment of each group is summarized in Table 1.

**Table 1:** Outline of Control and Treatment Groups.

Group:	Control Group (C)	Ribosylated Group (R)	Experimental Group 1 (X1)	Experimental Group 2 (X2)	Experimental Group 3 (X3)	Experimental Group 4 (X4)
1^st^ Treatment:	Buffered Saline Solution at 37°C for 7 days.	Buffered solution with 0.6 M Ribose at 37°C for 7 days.	Buffered solution with 0.6 M Ribose at 37°C for 7 days.	Buffered solution with 0.6 M Ribose at 37°C for 7 days.	Buffered solution with 0.6 M Ribose at 37°C for 7 days.	Buffered solution with 0.6 M Ribose at 37°C for 7 days.
2^nd^ Treatment:	Buffered PBS solution at 37°C for 7 days	Buffered PBS solution at 37°C for 7 days	PBS solution with 0.015 M PTB at 37°C for 3 days	PBS solution with 0.015 M PTB at 37°C for 7 days	PBS solution with 0.15 M PTB at 37°C for 3 days	PBS solution with 0.15 M PTB at 37°C for 7 days

A base solution for glycation was prepared in Hanks buffer (Sigma Inc. Ref# H9269) with a final concentration of 25 mM E-amino-n-caproic acid (Sigma Inc. Ref# A2504), 5 mM Benzamidine (Fluka Chemika Ref#12072), 10 mM N-ethylmaleimide (Sigma Inc. Ref# E3876-5G), and 30 mM of Hepes buffer (Sigma Inc. Ref# H3375). One-sixth of this solution was set aside for the control group. D-ribose (Sigma Inc. Ref# R 7500) was added to the remainder of the base solution to create a 0.6 M ribose solution.

The control specimens (C) were submersed in the control (non-ribose) solution while the remaining specimens (R, X1, X2, X3 and X4) were submersed in the ribose solution at 37°C. The temperature was maintained at 37°C and, if necessary, the pH of the solution was adjusted and maintained between 7.2 and 7.6 using 0.5N NaOH or 0.5N HCl. We have previously demonstrated that this incubation protocol does not cause loss of mineral content from bone [14], [17].

Two solutions of PTB [0.15 M and 0.015 M] were prepared in stock phosphate buffered saline (PBS). Experimental groups X1 and X2 were submersed in identical aliquots of 0.15 M PTB; the X1 for 3 days and the X2 for 7 days at 37°C. Experimental groups X3 and X4 were submersed in identical aliquots of 0.015 M PTB; the X3 for 3 days and the X4 for 7 days. The control and ribosylated groups were each submersed in a solution of PBS at 37°C. Since PTB has a short half-life in PBS [18], the PTB solution was replaced daily. The PBS solution was changed in the control and ribosylated groups every day as well. On the fourth day, experimental groups X1 and X3 were rinsed with PBS and were submersed in PBS in the same fashion as the control and ribosylated groups for the remainder of the seven days. At completion of the second treatment, all specimens were thoroughly rinsed with saline and subjected to mechanical testing.

### Mechanical Testing

Cancellous bone cylinders, subjected to chemical treatments described above, were glued to a pair of precision-machined brass end-caps and tested using a Mini-Bionix Servo-Hydraulic tensile testing machine (Model 858; MTS Systems). For testing, the machine's actuator was brought into contact with the specimen and the specimen was allowed to rest for 2 minutes while being irrigated with normal saline. The specimen was then compressed to failure at a constant strain rate of 0.02 mm/sec while measuring force and displacement at a rate of 100 Hz.

The test data were converted to stress and strain and used for obtaining the following variables. Young's modulus was determined using the eleven-point regression method from the linear portion of the curve [19]. Yield stress and yield strain were determined from the point where the stress-strain curve deviated outside of the 95% confidence interval of the linear portion of the curve. Ultimate stress and ultimate strain were defined by the maximum stress observed during the compression test. The secant modulus was calculated by dividing the ultimate stress by the ultimate strain. The strain ratio (Ultimate Strain/Yield Strain), and post yield strain (Ultimate Strain – Yield Strain) were also calculated for each specimen.

### AGE Analysis

After mechanical testing, each specimen was demineralized in 70% formic acid and complete demineralization was ensured by using a Poly No-Cal Endpoint Determination Kit (www.polysciences.com). One half of each demineralized bone specimen was papain digested in 0.4 mg/mL papain collagenase in 0.1 mM sodium acetate buffered to a pH of 6.0 for 16 hours at 65°C. AGE content was determined by fluorescence in an MR600 micro-plate reader using 370-nm excitation and 440-nm emission wavelength standardized against a serially-diluted quinine sulphate solution. The remaining halves of demineralized bone were dried. Collagen content was determined by hydroxyproline assay using hydrolysates of the papain digested specimens [14], [20].

### Statistics

Because AGE content data exhibited large variability across age groups, the data was parsed in two groups- young (19–49) and old (>50) to reduce the variance of the means. The young versus old cutoff age was set at 50 years old based on previous laboratory observations showing that younger bone contains lower levels of in vivo AGEs and is therefore more susceptible to in-vitro NEG than old bone [21]. As such, the “Old” specimens exhibited a smaller percentage increase in NEG from control to ribosylated groups.

A univariate analysis of variance (1-way ANOVA) was performed on the response variables (yield strain, ultimate strain, and post-yield strain) for each control and experimental group for every donor. Post-yield strain and strain ratio were normalized within each group and averaged and displayed with standard deviations. Significance for the null hypothesis as shown was determined by 1-way ANOVA at α = 0.05.

One-way ANOVA was selected over two-way because age was not considered a treatment variable. A student's t-test was only used when data was parsed into two paired groups of normalized data with equal variance. In that case, a two-tailed, paired, student's t-test was used.

## Results

### AGEs

**Figure 1:**
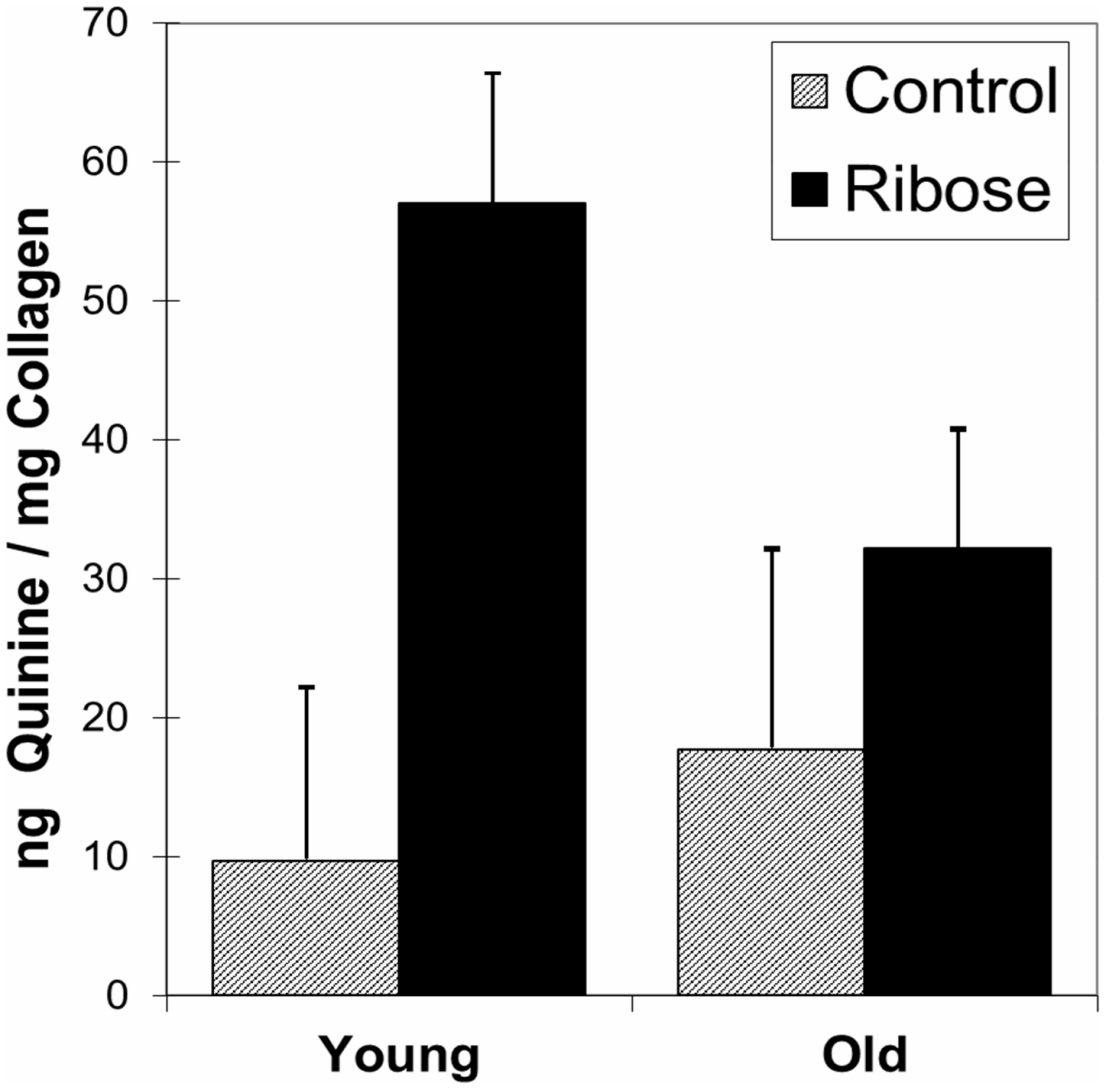
NEG Content for Control (shaded) and Ribosylated (solid black) specimens in Young and Old groups (p<0.05).

Presence of AGEs was observed as a uniform color change of the bone specimen from white to brownish-yellow (Figure 1). For both Old and Young groups, AGE content, defined as nanograms of quinine-sulphate fluorescence per milligram of collagen, increased markedly (p<0.05) from control to ribosylated groups. The Old Control group had more AGEs than the Young Control group (p<0.01) which verifies the presence and increase of in-vivo AGEs with age.

**Figure 2:**
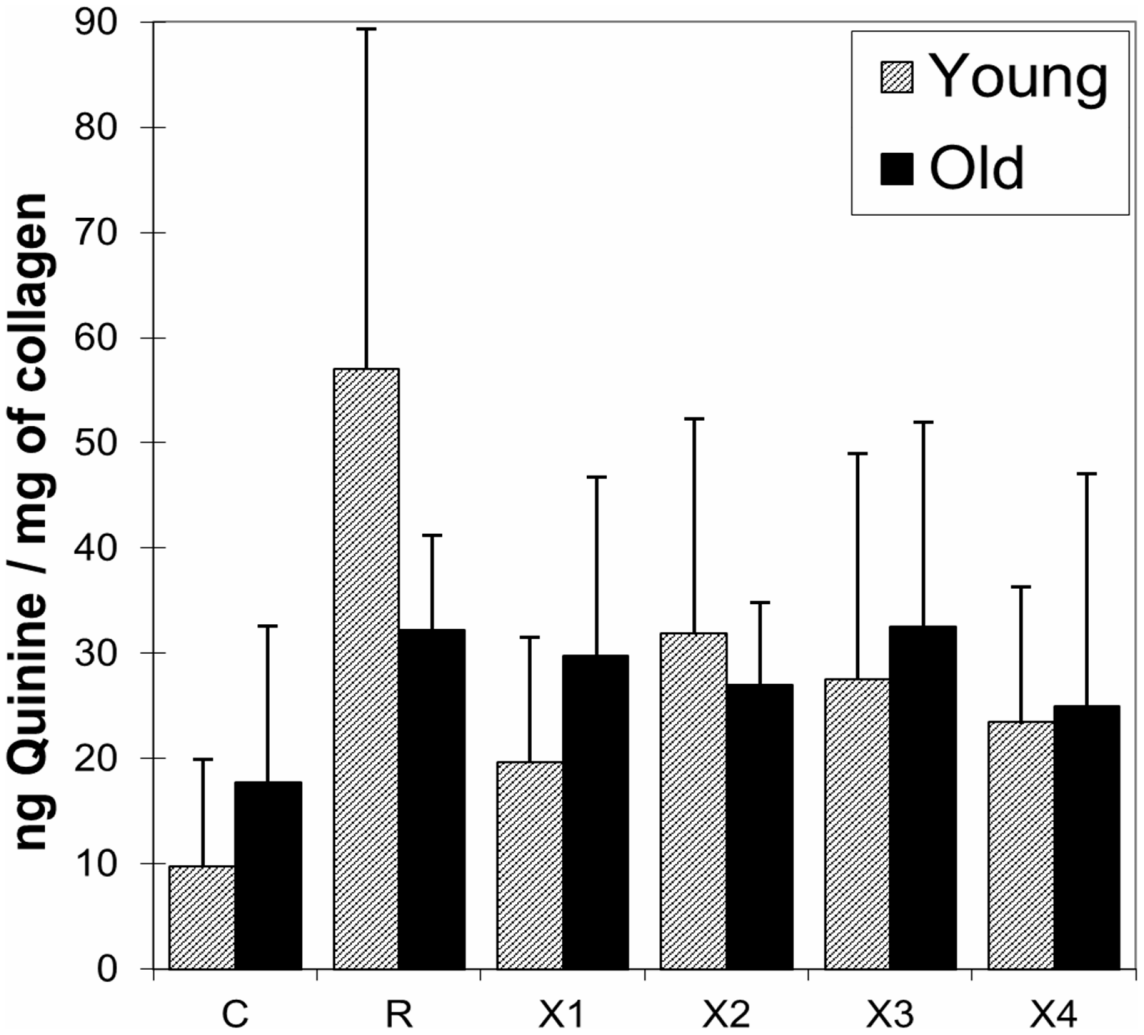
NEG content for Young (shaded) vs. Old (solid black) for all treatment groups C-control, R-ribosylated, X1 & X2 0.015 M PTB for 3 & 7 days, respectively and X3 & X4 0.15 M PTB for 3 & 7 days, respectively.

As shown in Figure 2, the AGEs for all treatment groups were decreased after PTB treatment. PTB treatment was more effective for specimens taken from younger donors, but it also significantly decreased AGEs in the Old group (p<0.05). There was no clear correlation between the amount of AGE reduction and PTB treatment concentration, but the seven day treatment resulted in a greater reduction of AGEs than the three day treatment.

### Mechanical Properties

**Figure 3:**
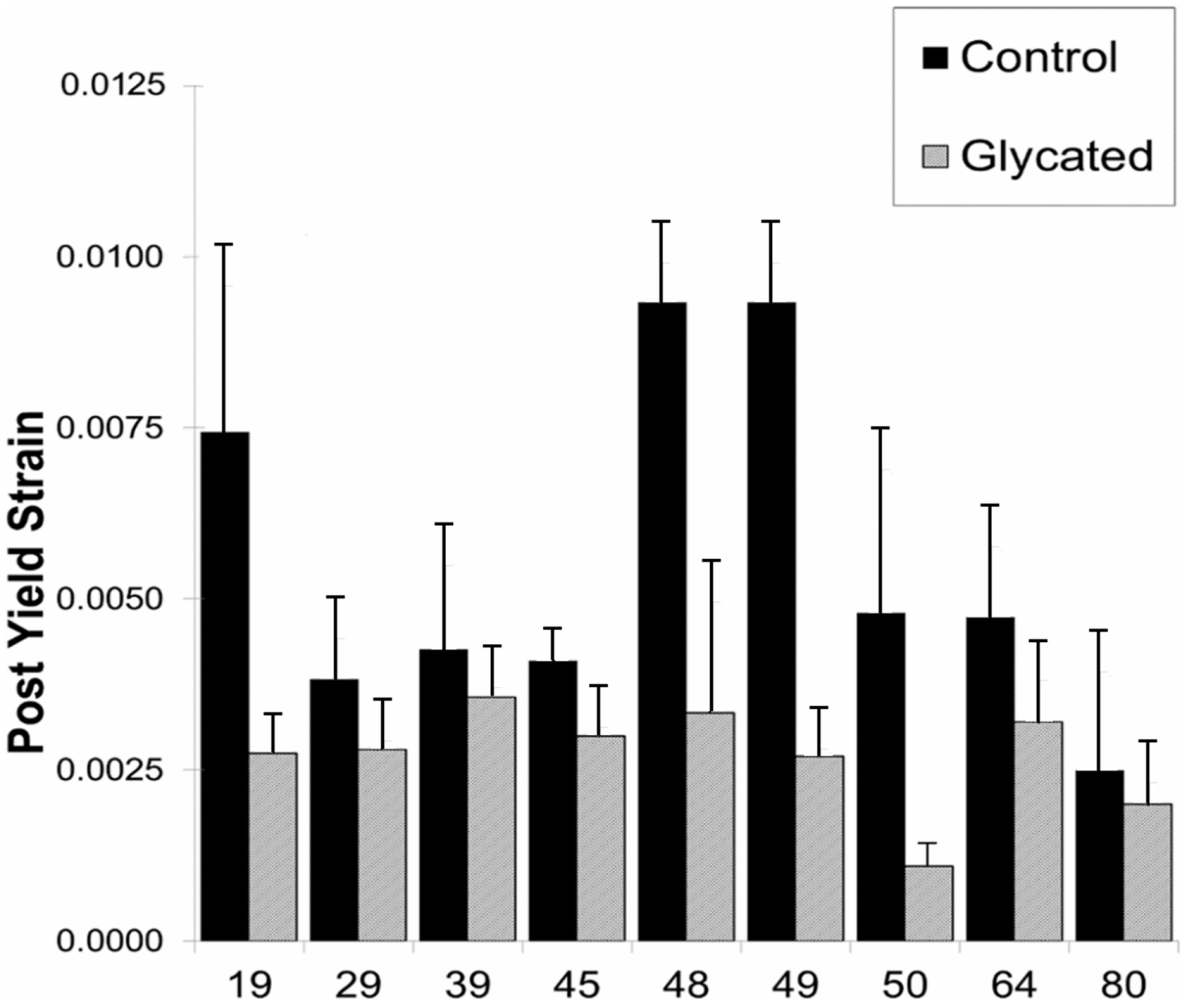
Post Yield Strain values of all donors are shown for Control specimens (solid black) against Ribosylated (grey) specimens. For all donors, post yield strain was significantly reduced (P<0.01).

**Figure 4:**
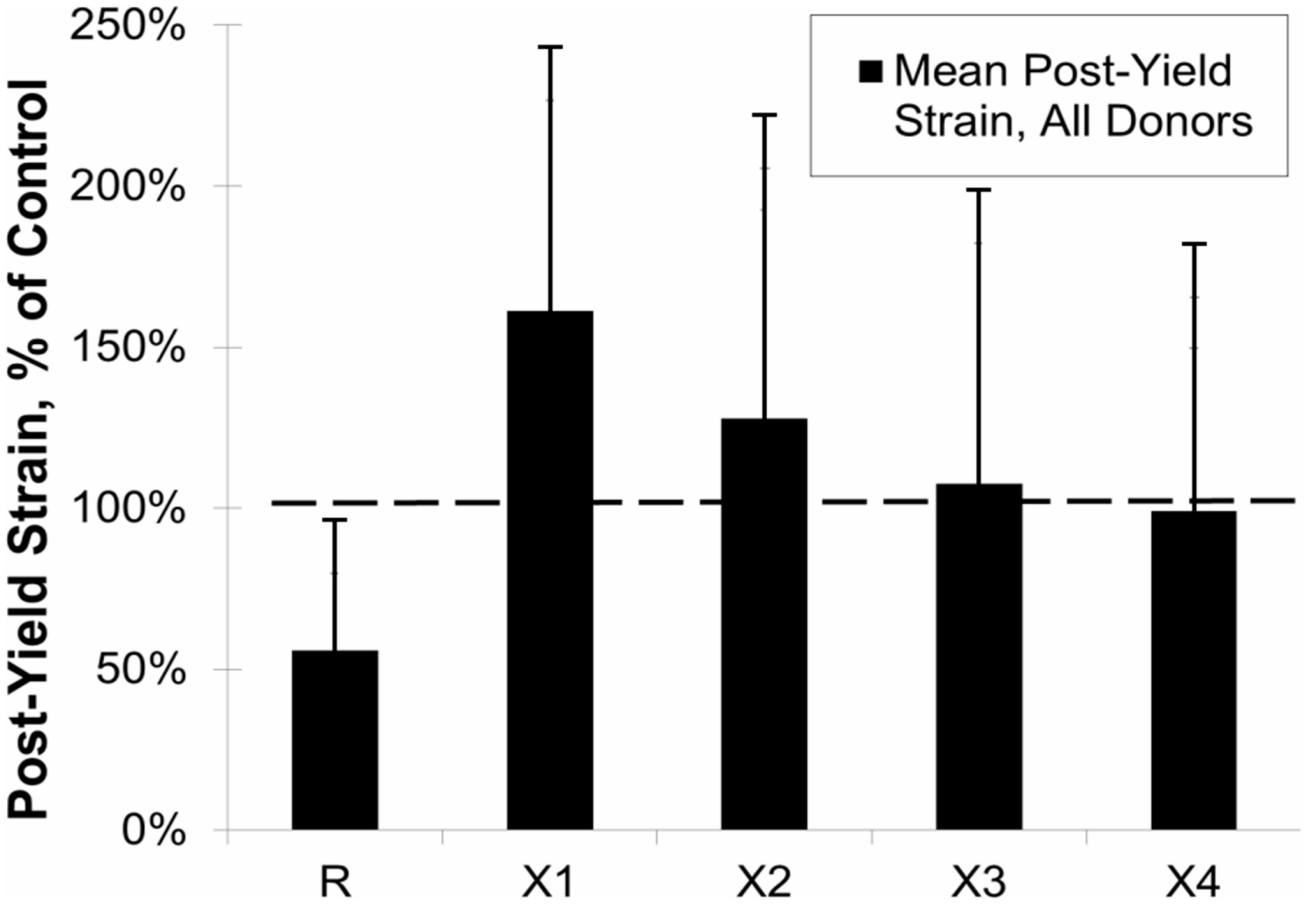
Effects of PTB on the Post-Yield Strain for all age groups is shown in black. R-ribosylated, X1 & X2 0.015 M PTB for 3 & 7 days, respectively and X3 & X4 0.15 M PTB for 3 & 7 days, respectively. Mean values of Post-Yield Strain were calculated and then normalized as a percentage of Control value. The dashed line represents control level. For all PTB treatments post-yield strain increased from the ribosylated value, (P<0.01).

Post-yield strain, an indicator of bone ductility, of Control and Ribosylated groups for all donors is shown in Figure 3. Ribosylated “R” groups exhibited a significant decrease in post-yield strain (p<0.01), which is consistent with brittle material behavior. The means for the normalized data from all donors are shown in Figure 4 as a percentage of control value. The PTB treatment groups X1-X4 show a significant increase of post-yield strain versus the ribosylated group back to control levels (p<0.01).

**Figure 5:**
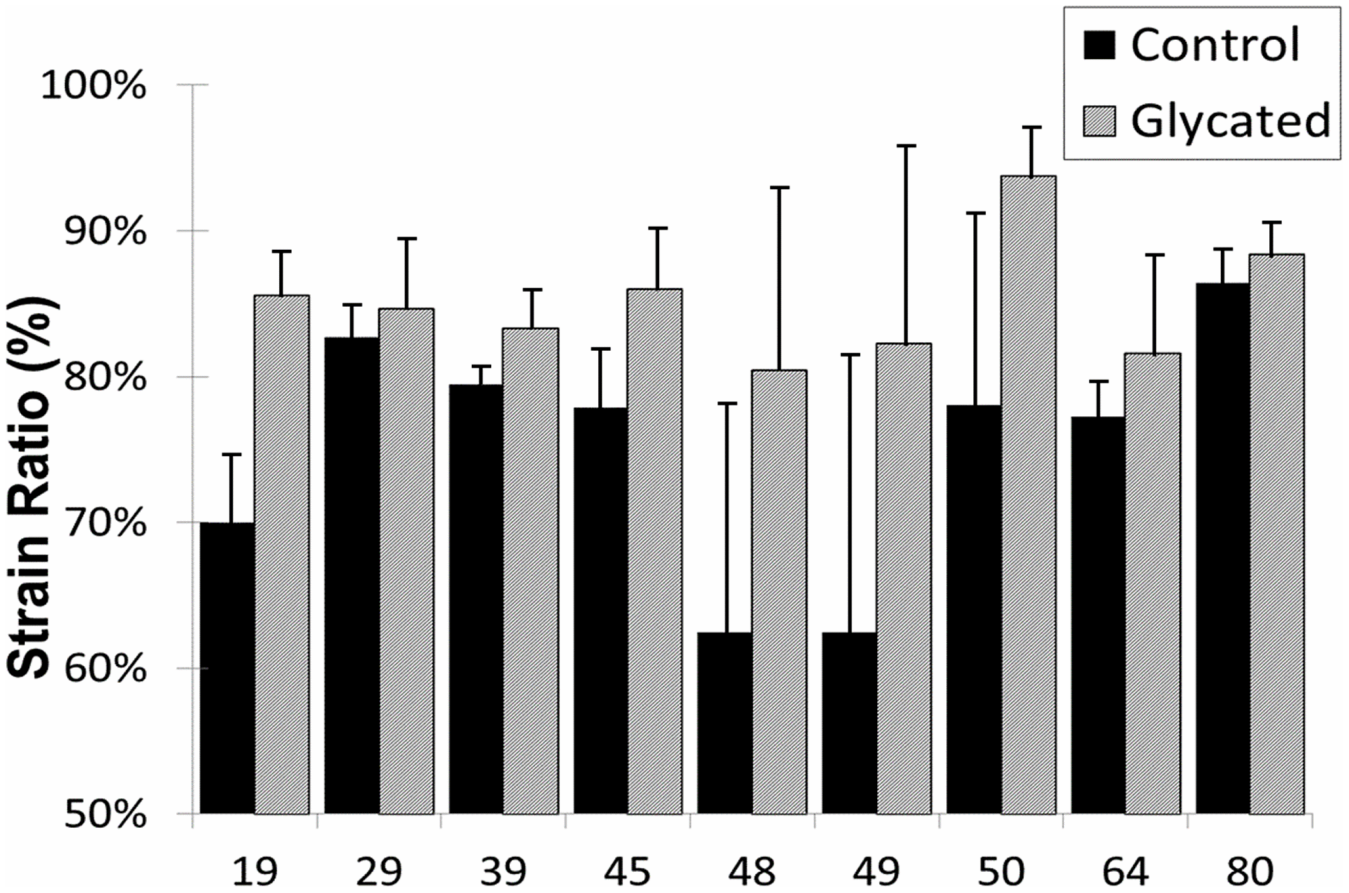
Strain Ratio values of all donors are shown for Control specimens (solid black) against Ribosylated (grey) specimens. For all donors, strain ratio was significantly increased (P<0.05) after glycation.

**Figure 6:**
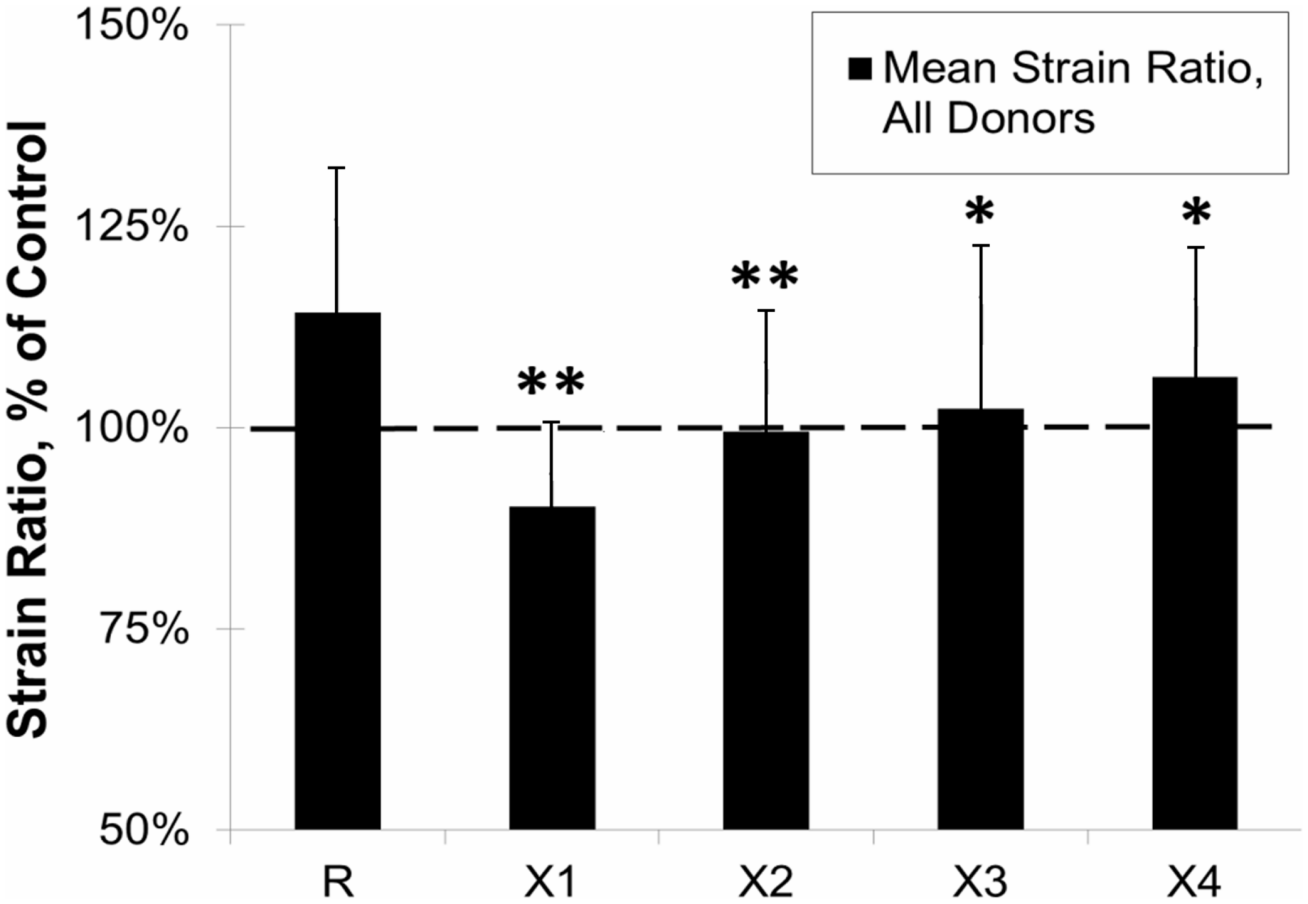
Effects of PTB on Strain Ratio for all age groups is shown in black. R-ribosylated, X1 & X2 0.015 M PTB for 3 & 7 days, respectively and X3 & X4 0.15 M PTB for 3 & 7 days, respectively. Mean values of Strain Ratio were calculated and then normalized as a percentage of Control value. The dashed line represents control level. For all PTB treatments strain ratio decreased from the ribosylated value, (*P<0.001, **P<0.05).

The strain ratio for each specimen, calculated by dividing ultimate strain by yield strain, represents the amount of energy dissipated pre- and post-yield and amplifies any significant changes in post-yield behavior. As shown in Figure 5, strain ratio was increased by glycation for all donors (p<0.05). Values for strain ratio were normalized against controls and the mean for each treatment group was calculated. Figure 6 shows the means for the PTB experimental groups (X1-X4) against the ribosylated group (R). For all treatment groups, strain ratio decreased toward control levels as represented by the dashed line. All treatment groups had a significant reduction in the strain ratio as compared to the ribosylated group (X1&X2 p<0.001, X3&X4 p<0.05).

## Discussion

One of the relevant age-related changes in bone quality is the accumulation of AGEs in bone's organic matrix [14], [17], [21]–[23]. At the macro-level, AGEs directly affect bone by reducing its post-yield material properties [17], [21]. On a micro-level, AGEs indirectly affect bone by slowing the remodeling process, allowing micro-damage and AGEs to accumulate at a greater rate [21], [24]–[26]. Here for the first time, we show the ability of n-Phenacylthiazolium Bromide to improve the quality of existing bone matrix by cleaving established AGE crosslinks known to accumulate with aging, disease, and the anti-resorptive treatment of osteoporosis.

PTB was originally identified as a potential crosslink breaker because of the susceptibility of AP-ene-dione-derived protein crosslinks to cleavage by certain thiazolium salts [16], [27]. PTB consists of nucleophilic centres at the thiazolium-2 position and the α-position of the N-substituent. These nucleophilic centres react with the carbonyl groups of an AGE crosslink to form a five-membered ring and thereby convert the carbonyl-group carbons to a tetrahedral geometry which facilitates spontaneous cleavage at physiological pH. When applied in practice, PTB was shown to selectively cleave established AGE crosslinks in vitro and in vivo by Vasan et al. [16]. Using their work as a baseline, we applied PTB to human bone previously subjected to non-enzymatic glycation. This resulted in a decrease in total AGE content, demonstrating the viability of PTB treatment for both old and young bone (Figure 2).

Post yield deformation is an important aspect of bone fracture specific to energy dissipation before failure. The amount of energy a bone can absorb before it breaks is dependent on its ability to first elastically deform (pre-yield), then to plastically deform in an effort to dissipate energy (post-yield) before ultimate failure. Glycation, both in vivo and in vitro, does not significantly alter pre-yield mechanics. However, it reduces the amount of energy the bone can dissipate post-yield and thereby increases fracture risk for otherwise benign traumas [14], [17], [21]. When alterations in post-yield material properties are combined with decreased bone mass (osteoporosis), the risk of fracture is further amplified. Results of this study show that the removal of AGE crosslinks using PTB restores bone's post-yield properties and reduces fragility. Furthermore, we found that PTB treatment is effective across age groups which suggests that PTB is able to cleave AGE crosslinks in older and more mature bone tissue.

The ability of PTB to reverse AGE accumulation in bone is also important because of the interaction between AGEs and the natural remodeling process. Recent research has shown that glycated bone is less susceptible to osteoclastic digestion and consequently has a slower resorption rate than normal bone [23]. Thus, decreasing the AGE content of mature bone may initiate bone turnover and promote new bone formation. This finding is especially relevant for patients undergoing anti-resorptive therapies including bisphosphonate (BP) therapy. Since bone resorption is reduced, AGEs will accumulate at a faster rate with BP therapy [15], [24]. If BP treatment is halted, or if an anabolic treatment like PTH is administered, the response of bone to the remodeling stimulus may still be reduced due to the increased AGE content and the inhibition of osteoclastic activity. PTB treatment during drug holidays, which are recommended throughout BP therapy, may improve bone quality in patients undergoing long term BP therapy or patients transitioning to PTH from BP [28], [29].

The major limitation of the work presented is that it was conducted in vitro. In order to prove feasibility for human use, in-vivo animal and human studies need to be performed. It is noteworthy that PTB has previously been tested in an in vivo animal model and successfully cleaved AGE crosslinks in soft-tissue [16], [27]. In light of these previous studies and the in-vitro results presented in this study, we suggest that PTB or its derivatives may be suitable treatments for improving bone quality and warrants further investigation.

In summary, this study demonstrates that AGEs crosslinks in human bone are susceptible to cleavage by a thiazolium-based nucleophile and that reducing the AGE content in bone restores the material-level post-yield properties of the trabecular architecture. With this discovery, a pharmacological intervention for age-related changes in bone's organic structure may be possible.
